# Epigenetic repression of Krüppel-like factor 4 through Dnmt1 contributes to EMT in renal fibrosis

**DOI:** 10.3892/ijmm.2015.2189

**Published:** 2015-04-20

**Authors:** XIANGCHENG XIAO, WENBIN TANG, QIONGJING YUAN, LING PENG, PINGPING YU

**Affiliations:** 1 Department of Nephrology, Xiangya Hospital, Central South University, Changsha, Hunan 410000, P.R. China; 2 The Nephrotic Laboratory of Xiangya Hospital, Central South University, Changsha, Hunan 410000, P.R. China; 3 Department of Geriatrics, Xiangya Hospital, Central South University, Changsha, Hunan 410000, P.R. China

**Keywords:** kidney disease, renal fibrosis, epithelial-to-mese-nchymal transition, Krüppel-like factor 4, hypermethylation

## Abstract

Krüppel-like factor 4 (KLF4) is a transcription factor which plays divergent roles in a number of physiological or pathological process. However, the expression and role of KLF4 in renal fibrosis remain undetermined. The aim of the present study was to determine the epigenetic alterations of KLF4 and its potential role and mechanisms of action in epithelial-to-mesenchymal transition (EMT) in renal fibrosis. The hypermethylation of the KLF4 promoter accompanied by a decrease in KLF4 expression were observed in mice subjected to unilateral ureteral obstruction (UUO) and in HK-2 cells stimulated with transforming growth factor (TGF)-β1. However, treatment with 5-aza-2′-deoxycytidine attenuated the TGF-β1-induced downregulation of KLF4 and E-cadherin and the upregulation of α-smooth muscle actin (α-SMA) in the HK-2 cells. DNA methyltransferase 1 (Dnmt1) participated in the TGF-β1-mediated hypermethylation of the KLF4 promoter in the HK-2 cells. In addition, functional analysis demonstrated that the overexpression of KLF4 led to an increase in the expression of E-cadherin and zonula occludens-l (ZO-1), and a decrease in the expression of α-SMA and fibroblast-specific protein 1 (FSP-1), thus reversing the effects of the suppression of KLF4. These data suggest that KLF4 inhibits the progression of EMT in renal epithelial cells. In conclusion, our findings demonstrate that KLF4 is downregulated during EMT in renal fibrosis *in vivo* and *in vitro*; thus, KLF4 functions as a suppressor of renal fibrogenesis. The hypermethylation of KLF4 directly mediated by Dnmt1 contributes to the progression of EMT in renal epithelial cells. KLF4 promoter methylation may thus be a promising diagnostic marker or therapeutic target in renal fibrosis.

## Introduction

Renal fibrosis is characterized by an enhancement fibroblast proliferation and an increase in matrix protein expression along with the loss of functioning nephrons. The final common outcome of renal fibrosis is generally tubulointerstitial fibrosis. It has been demonstrated that epithelial-to-mesenchymal transition (EMT) is involved in the pathogenesis of tubulointerstitial fibrosis (1). In this process, tubular epithelial cells acquire a mesenchymal phenotype which results in their transition into myofibroblasts (2,3).

It is currently well known that EMT plays an important role in embryonic development, as well as in wound healing, tissue regeneration, cancer progression and organ fibrosis, including renal fibrosis and is regulated by a series of transcription factors and signaling pathways (4–7). It has been demonstrated that the EMT-mediated renal fibrosis is affected by a number of factors, including transforming growth factor (TGF)-β1, connective tissue growth factor (CTGF), Angiotensin II (ANG II), fibroblast growth factor (FGF)-2, interleukin (IL)-1, hepatocyte growth factor (HGF) and bone morphogenetic protein 7 (BMP-7) (8). The zinc-finger transcription factor, Krüppel-like factor 4 (KLF4), performs divergent functions in a number of physiological or pathological process. Recent studies have demonstrated that KLF4 expression is decreased or lost in cancer and the inhibition of its expression induces EMT by regulating the expression of EMT-related genes, such as E-cadherin, N-cadherin, vimentin, β-catenin, vascular endothelial growth factor (VEGF)-A, endothelin-1 and Jnk1 in breast cancer (9) or through crosstalk with the TGF-β, Notch and Wnt signaling pathways in gastrointestinal cancer (10). Moreover, it has been reported that KLF4 is epigenetically silenced by promoter methylation and functions as a tumor suppressor gene in renal cell carcinoma (11). However, the expression pattern and role of KLF4 during EMT in renal fibrosis remain unclear.

Epigenetic modificaions are essential for the development and function of the kidneys (12), and aberrant methylation (13), histone modifications or the dysregulation of microRNAs may cause chronic kidney disease (14–16). It has been demonstrated that the hypermethylation of RASAL1, encoding an inhibitor of the Ras oncoprotein, is mediated by DNA methyltransferase 1 (Dnmt1) and is associated with fibrogenesis in the kidneys (17). This prompted us to hypothesize that the epigenetic repression of KLF4, similar to its role in renal cell carcinoma (11), may promote EMT in renal fibrogenesis.

In the present study, the expression, methylation status and function of KLF4 in renal fibrosis were investigated. To the best of our knowledge, we demonstrate for the first time that the hypermethylation of KLF4 is associated with EMT in renal fibrosis and that this hypermethylation is regulated by Dnmt. Thus, our findings provide a new molecular basis for EMT in epithelial cells in renal fibrosis.

## Materials and methods

### Cell culture

Human renal proximal tubule epithelial cells (HK-2 cells, ATCC^®^ CRL-2190) were cultured in RPMI-1640 medium containing 2,000 mg/l NaHCO_3_, supplemented with 10% fetal bovine serum (FBS), 100 U/ml penicillin and 100 *μ*g/ml streptomycin in 5% CO_2_ at 37°C. The cells were then stimulated for different periods of time with human recombinant TGF-β1 (1 ng/ml; PeproTech, Rocky Hill, NJ, USA). Cell culture reagents were obtained from Life Technologies Inc., Carlsbad, CA, USA.

### Animal model of unilateral ureteral obstruction (UUO)

Male CD-1 mice were obtained from the Xiangya Hospital of Central South University, Changsha, China. The mice in the present study were divided into 3 groups as follows: i) the mice in the control group (Con) were fed without surgical manipulation; ii) the mice in the sham-operated group were subjected to surgical manipulation without ureteral ligation; and iii) the mice in the unilateral ureteral obstruction (UUO) group were subjected to left ureteral ligation. UUO was performed as previously described (18). All mice were sacrificed 14 days following UUO and the kidney tissues were collected for various analyses. The protocols for animal experimentation and maintenance were approved by the Animal Ethics Committee at our institute and carried out in accordance with institutional guidelines.

### Methylation-specific PCR (MSP)

MSP was carried out on bisulfate-treated DNA using the EZ DNA Methylation-Gold™ kit (Zymo Research, Irvine, CA, USA) according to the manufacturer’s instructions. Methylation-specific primers for the KLF4 promoter were designed using the MethPrimer program, as previously described (19). Bisulfite converted genomic DNA was PCR-amplified using methylation-specific primers and SYBR-Green reaction mix. The human primers used were unmethylated and were as follows: KLF4 forward, 5′-tgtagggtttaaataggtgataatga-3′ and reverse, 5′-aaataataaaaa ctcaaacaccaaa-3′; and methylated KLF4 forward, 5′-cgta gggtttaaataggtgataacg-3′ and reverse, 5′-aaataataaaaactcg aacaccgaa-3′. The mouse primers used were unmethylated and were as follows: KLF4 forward, 5′-ttgtaaagagttttgagatttttgg-3′ and reverse, 5′-caaaattaaacacataatcatcatt-3′; and methylated KLF4 forward, 5′-gttcgtaaagagttttgagattttc-3′ and reverse, 5′-gaaattaaacacgtaatcatcgtt-3.

### Epigenetic drug treatment of the cells

The cells were treated with the demethylation drug, 5-Aza-2′-deoxycytidine (5-Aza), which was purchased from Sigma-Aldrich (St. Louis, MO, USA; A3656) at 15.5 nM for 72 h.

### Reverse transcription-quantitative PCR (RT-qPCR)

The total RNA was extracted from the cells using TRIzol reagent (Invitrogen, Carlsbad, CA, USA) according to the manufacturer’s instructions, and the RNA was subsequently reverse transcribed using the PrimeScript RT Master Mix Perfect Real-Time kit (Takara, Dalian, China) to obtain the cDNA. Using the cDNA as a template, a real-time PCR assay was performed using the following pairs of primers: KLF-4 forward, 5′-ctgagcagcagggactg-3′ and reverse, 5′-ttgagatgggaactctttgt-3′ in HK-2 cells, and KLF-4 forward, 5′-ttctccacgttcgcgtccgg-3′ and reverse, 5′-tctcgccaacggttagtcgggg-3′ in mice; and Dnmt1 forward, 5′-aaccttcacctagccccag-3′ and reverse, 5′-tgacaggtggt cactcctcatg-3′; Dnmt3A forward, 5′-tattgatgagcgcacaagagagc-3′ and reverse, 5′-gggtgttccagggtaacattgag-3′; Dnmt3B forward, 5′-tacacagacgtgtccaacatgggc-3′ and reverse, 5′-gaggaagctgct aaggactagttc-3′; E-cadherin forward, 5′-aatgccgccatcgcttacac-3′ and reverse, 5′-cgacgttagcctcgttctca-3′; α-smooth muscle actin (α-SMA) forward, 5′-tcccttgagaagagttacgagtt-3′ and reverse, 5′-catgatgctgttgtaggtggtt-3′; zonula occludens-l (ZO-1) forward, 5′-ggaagttacgtggcgaag-3′ and reverse, 5′-ctctggcggacatcttgt-3′; fibroblast-specific protein 1 (FSP-1) forward, 5′-cctctctacaa ccctctct-3′ and reverse, 5′-ggacaccatcacatccag-3′; and β-actin forward, 5′-aggggccggactcgtcatact-3′ and reverse, 5′-ggcggcacc accatgtaccct-3′. The 20 *μ*l real-time PCR reaction included 0.5 *μ*l of cDNA template, 0.25 *μ*l of Primer F, 0.25 *μ*l of Primer R, 10 *μ*l of RNase-free dH_2_O and 8 *μ*l of 2.5X Real-Time Master Mix (SYBR-Green I). The reaction conditions included a pre-denaturation step at 95°C for 10 sec, and 40 cycles of 95°C for 15 sec and 60°C for 60 sec. After the reaction, the data were subjected to statistical analysis.

### Construction of lentivirus (LV) for KLF4 overexpression

To generate the KLF4-overexpression plasmid, KLF4-CDS (NM_004235.4) was amplified by PCR using the following primers: forward, 5′-**gaattca**tgaggcagccacctggc-3′ (including the *Eco*RI site, shown in bold font) and reverse, 5′-**ggatcc**ttaaaaat gcctcttcatgtg-3′, (including the *Bam*HI site, shown in bold font); the sequence was then cloned into the *Bam*HI/*Eco*RI sites of the pLV-Neo vector (Inovogen, Beijin, China). The pLV-Neo-KLF4 or pLV-Neo vectors were co-transfected with packaging plasmids into 293T cells using Lipofectamine 2000. The viral supernatant of pLV-Neo-KLF4 or pLV-Neo was filtered through a 0.45-*μ*m filter and renamed as Lv-KLF4 and Lv-Con, respectively. All the supernatants were used to infect the target cells with 10 *μ*g/ml of polybrene (Sigma-Aldrich). After the target cells were infected for 96 h, KLF4 expression was confirmed by quantitative (real-time) PCR and western blot analysis (Fig. 3). The cells infected with Lv-KLF4 and Lv-Con were used for further analyses.

### Chromatin immunoprecipitation (ChIP) assay

ChIP assay was carried out as previously described (20). ChIP assays were performed using the ChIP-IT kit (Active Motif, Carlsbad, CA, USA) according to the manufacturer’s instructions. The HK-2 cells were conventionally cultured to approximately 70–80% confluency, the medium was decanted, and the medium containing 1% formaldehyde was then added to fix the cells. The cells were washed with cold PBS, and then glycine was added to terminate the fixation, and the cells were washed again with cold PBS. After the cells were scaped from the culture plate, the cold cell lysate was centrifuged to collect the nuclei. The pelleted nuclei were resuspended, and chromatin was cleaved into approximately 500-bp fragments by ultrasonication. The cleaved chromatin was treated with RNase and proteinase K, and the extent of cleavage was then assessed by 1% agarose gel electrophoresis. Protein G beads were used to remove the non-specific antibodies from the chromatin preparation, Subsequently, an anti-Dnmt1 antibody (Cat. no. ab13537; Abcam, Cambridge, UK), a positive control RNA polymerase (pol) II antibody (Cat. no. ab5131; Abcam) and a negative control IgG antibody (Cat. no. ab2410; Abcam) were added. The cells were incubated overnight at 4°C. Protein G beads were added to the solution containing the antibody/chromatin complexes, which were then washed and eluted, de-cross-linked and the co-precipitated DNA was purified. Finally, the co-precipitated DNA was used as a template in a PCR amplification using the following primers: forward, 5′-cag gagaatcgcttggacg-3′ and reverse, 5′-gaacaggcggaaagaggc-3′.

### Western blot analysis

The cells were lysed in cell lysate, and then centrifuged at 12,000 × g for 20 min at 4°C. The supernatant was collected and denatured. Proteins were separated by 10% SDS-PAGE and blotted onto polyvinylidene difluoride (PVDF) membranes. The PVDF membranes were treated with TBST containing 50 g/l skimmed milk at room temperature for 4 h, followed by incubation with the primary antibodies, anti-KLF4 (Cat. no. BM0485; Abzoom Biolabs, Dallas, TX, USA), anti-E-cadherin (Cat. no. BM0530; Abzoom Biolabs), anti-α-SMA (Cat. no. YT5053; ImmunoWay Biotechnology Company, Newark, DE, USA), anti-ZO-1 (1:1,000 dilution; Cat. no. ab59720; Abcam), anti-FSP-1 (1:5,000 dilution; Cat. no. ab27957; Abcam) and anti-β-actin (Cat. no. BM0272; Abzoom Biolabs), respectively, at 37°C for 1 h. The membranes were rinsed and incubated for 1 h with the corresponding peroxidase-conjugated secondary antibodies. Chemiluminescent detection was performed using the ECL kit (Pierce Chemical Co., Rockford, IL, USA). The amount of the protein of interest, expressed as arbitrary densitometric units, was normalized to the densitometric units of β-actin.

### Immunocytochemistry

The expression of E-cadherin, ZO-1, α-SMA and FSP-1 in the control HK-2 cells (infected with Lv-Con) and stimulated or not with TGF-β1, and in the HK-2 cells infected with Lv-KLF4 and stimulated with TGF-β1, was determined by immunocytochemistry. A standard immunostaining procedure was performed using antibodies against E-cadherin (1:500 dilutions), α-SMA (1:500 dilutions), FSP-1 (1:200 dilutions), ZO-1 (1:100 dilutions). Protein expression was graded on a scale of ‘±’ to ‘+++’ as previously described (21).

### Statistical analysis

Data are expressed as the means ± SD from at least 3 separate experiments. Statistical analysis was carried out using SPSS 15.0 software. Differences between 2 groups were analyzed using the Student’s t-test. A value of P<0.05 was considered to indicate a statistically significant difference.

## Results

### The expression and methylation status of KLF4 in mice subjected to UUO

**Figure 1:**
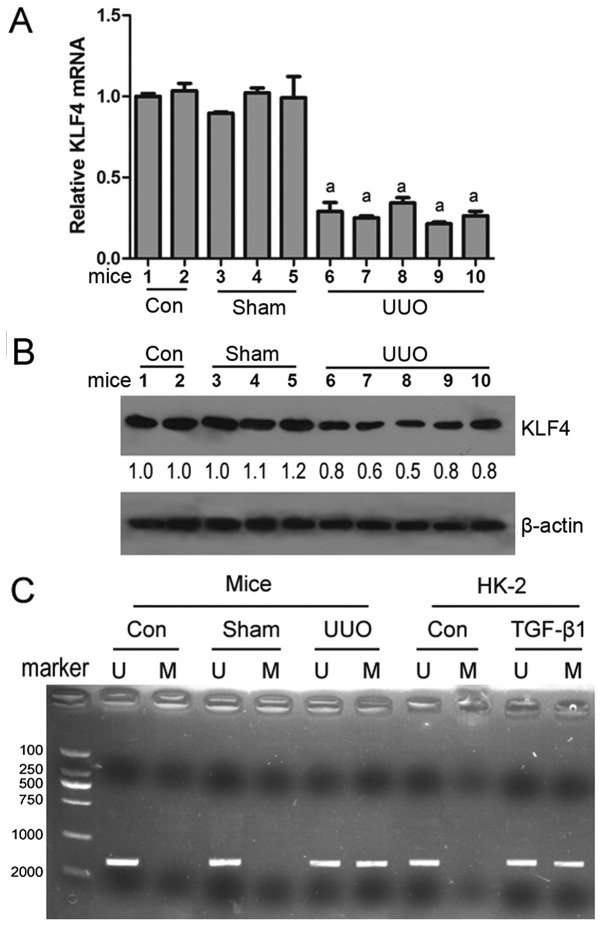
Expression and methylation status of Krüppel-like factor 4 (KLF4) in mice subjected to unilateral ureteral obstruction (UUO) or in HK-2 cells stimulated with transforming growth factor (TGF)-β1. KLF4 (A) mRNA and (B) protein expression was decreased in renal tissues of mice subjected to UUO compared with the mice in the control (Con) or sham-operated (Sham) group. ^a^P<0.05 vs. Con or Sham group. (C) Methylation status of the KLF4 promoter was determined by PCR using specific primers. M, methylation; U, unmethylation. ^a^P<0.05 vs. Con (no treatment).

To determine whether KLF4 is involved in renal fibrosis, an animal model of renal disease was created by subjecting mice to UUO. RT-qPCR and western blot analysis revealed that the KLF4 mRNA and protein levels were decreased in the renal tissues from the mice subjected to UUO compared with the mice in the control or sham-operated groups (Fig. 1A and B). MSP analysis revealed that KLF4 methylation was observed in the renal tissues of mice subjected to UUO, while KLF4 methylation was not observed in the mice in the control group or sham-operated group (Fig. 1C). These results suggest that KLF4 is downregulated by hypermethylation, thus contributing to renal fibrogenesis.

### KLF4 is hypermethylated and downregulated in HK-2 cells stimulated with TGF-β1

**Figure 2:**
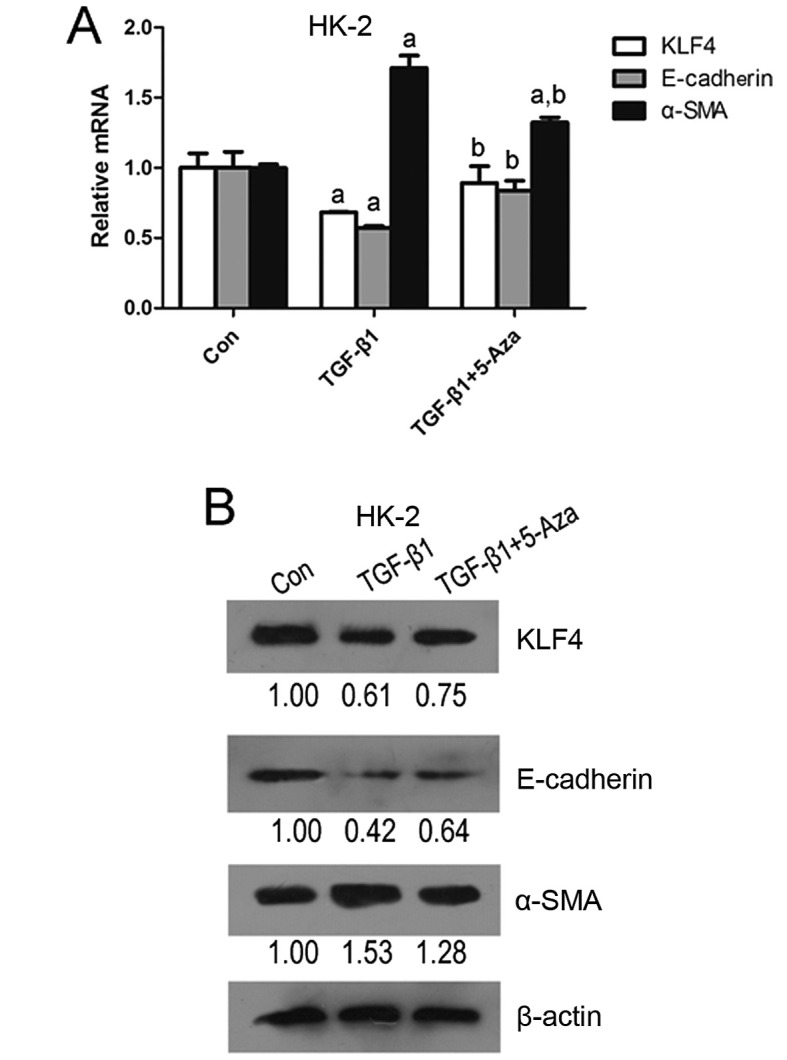
Expression of Krüppel-like factor 4 (KLF4), E-cadherin and α-SMA in HK-2 cells. The relative (A) mRNA and (B) protein expression of KLF4, E-cadherin and α-smooth muscle actin (α-SMA) in HK-2 cells stimulated with transforming growth factor (TGF)-β1 or TGF-β1 + 5′-aza-2,-deoxycyt-idine (5-Aza) was determined by RT-qPCR or western blot analysis. ^a^P<0.05 vs. control (Con) group; ^b^P<0.05 vs. treatment with TGF-β1 alone.

To further determine whether KLF4 is involved in renal fibrosis by affecting EMT in renal tubular epithelial cells, human renal proximal tubule epithelial cells (HK-2 cells) were stimulated with TGF-β1, which induces EMT in epithelial cells. Following stimulation for 36 h, a decrease in KLF4 and E-cadherin expression accompanied by an increase in α-SMA expression were observed in the HK-2 cells (Fig. 2), suggesting that KLF4 functions as a suppressor of EMT induced by TGF-β1 in tubular epithelial cells. Moreover, 5-aza-2′-deoxycytidine, as a demethylating agent, attenuated the TGF-β1-induced downregulation in KLF4 expression (Fig. 2). MSP analysis indicated that both methylated and unmethylated KLF4 were detected in the HK-2 cells stimulated with TGF-β1; however, only unmethylated KLF4 was detected in the controls (untreated cells) (Fig. 1C). These results demonstrate that the downregulation of KLF4 is at least partly induced by hypermethylation alternation, and that this may promote EMT in tubular epithelial cells in the process of renal fibrogenesis.

### Dnmt1 is directly involved in the hypermethylation of KLF4 in HK-2 cells stimulated with TGF-β1

**Figure 3:**
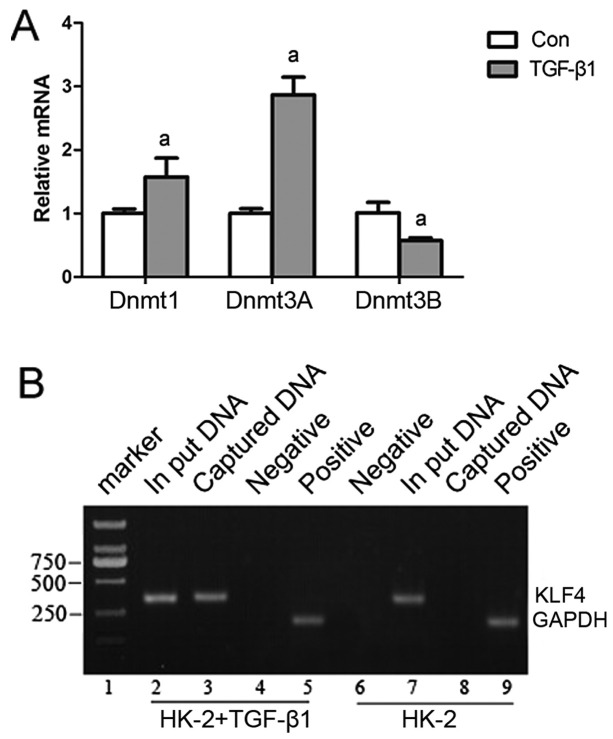
DNA methyltransferase 1 (Dnmt1) is involved in the methylation of Krüppel-like factor 4 (KLF4) in HK-2 cells stimulated with transforming growth factor (TGF)-β1. (A) An increased Dnmt1 and Dnmt3A and a decreased Dnmt3 expression was detected in the HK-2 cells stimulated with TGF-β1 by RT-qPCR. (B) ChIP assay shows the specific segments in the KLF4 promoter detected in the chromatin immunoprecipitation complex captured by specific antibodies to Dnmt1.

The results from RT-qPCR revealed that the Dnmt1 and Dnmt3A expression levels were significantly increased in the HK-2 cells stimulated with TGF-β1; however, Dnmt3B expression was decreased (Fig. 3A). ChIP assay indicated that after Dnmt1 protein-KLF4 promoter complexes were captured with Dnmt1-specific antibodies, KLF4-promoter-specific segments were detected (Fig. 3B). These data demonstrate that Dnmt1 plays a direct role in the TGF-β1-mediated hypermethylation of KLF4 in HK-2 cells.

### KLF4 functions as a suppressor of EMT in renal epithelial cells

**Figure 4:**
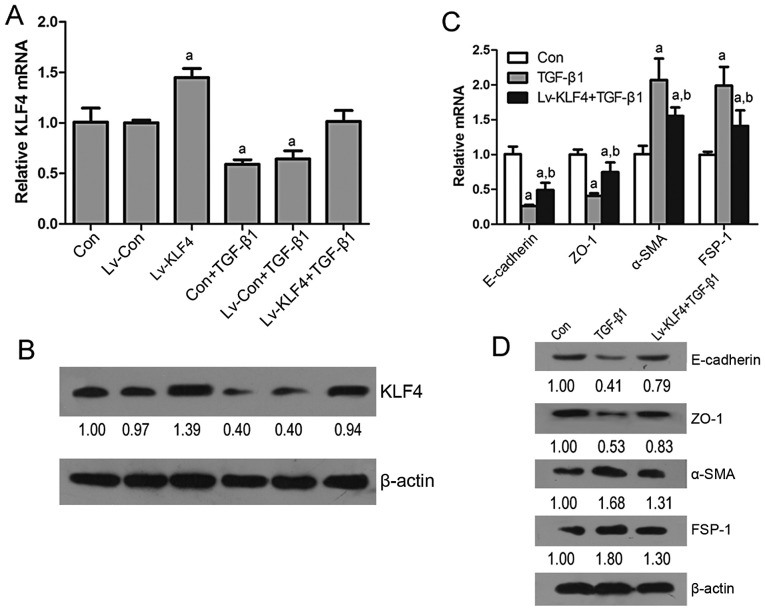
Krüppel-like factor 4 (KLF4) functions as a suppressor of epithelial-to-mesenchymal transition (EMT) in renal epithelial HK-2 cells. Relative (A) mRNA and (B) protein expression of KLF4 in HK-2 cells treated with Lv-KLF4 or transforming growth factor (TGF)-β1 or with Lv-KLF4 + TGF-β1 was determined by RT-qPCR or western blot analysis. Relative (C) mRNA and (D) protein expression of E-cadherin, zonula occludens-l (ZO-1), α-smooth muscle actin (α-SMA) and fibroblast-specific protein 1 (FSP-1) in HK-2 cells treated with TGF-β1 or Lv-KLF4 + TGF-β1. ^a^P<0.05 vs. control (Con) group; ^b^P<0.05 vs. treatment with TGF-β1 alone.

**Figure 5:**
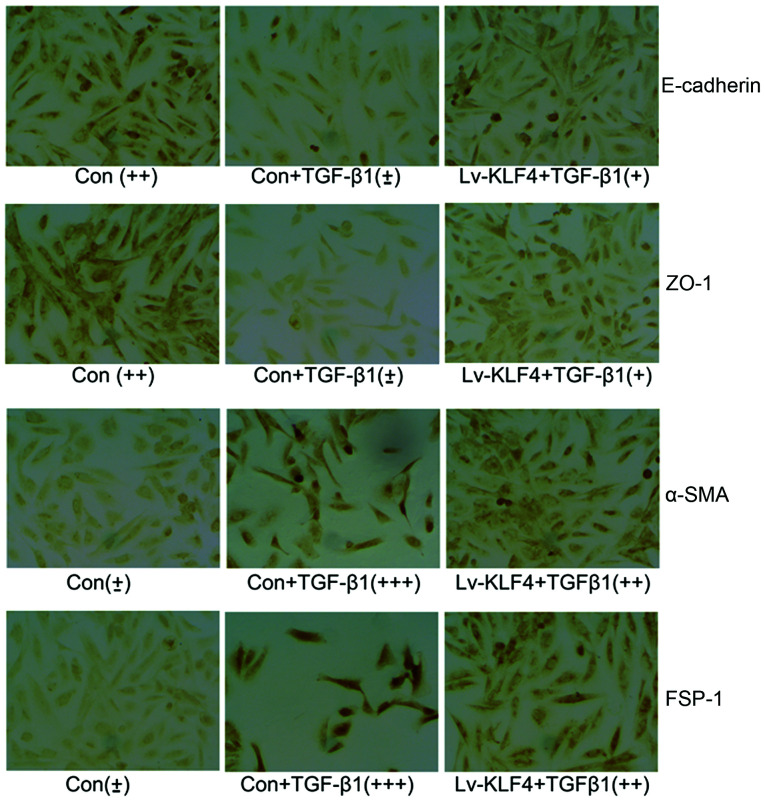
Immunocytochemical staining showing the expression of epithelial-to-mesenchymal transition (EMT) markers. E-cadherin, zonula occludens-l (ZO-1), α-smooth muscle actin (α-SMA) and fibroblast-specific protein 1 (FSP-1) in HK-2 cells treated with TGF-β1 or Lv-KLF4 + TGF-β1 using the antibodies respective. Protein expression was graded on a scale of ‘±’ to ‘+++’.

To validate whether KLF4 plays a role in EMT in renal epithelial cells, the expression of KLF4 was enforced in the HK-2 cells. The results from RT-qPCR and western blot analysis revealed that the transfection of the HK-2 cells with Lv-KLF4 induced an increase in the expression of KLF4, E-cadherin and ZO-1, and a decrease in the expression of α-SMA and FSP-1, and that this attenuated the TGF-β1-induced downregulation in KLF4, E-cadherin and ZO-1 expression and the upregulation in α-SMA and FSP-1 expression (Fig. 4). In addition, immunocytochemistry of the HK-2 cells infected with Lv-KLF4 or Lv-KLF4 + TGF-β1 revealed similar results as regards the expression of these factors (Fig. 5). These data confirm that KLF4 inhibits the progression of EMT induced by TGF-β1 in renal epithelial cells.

## Discussion

EMT in the adult kidneys is one of the key events in renal tubulointerstitial fibrosis (22). The transition of tubular epithelial cells into myofibroblasts through EMT finally contributes to renal fibrogenesis. Strutz *et al* (23) discovered that tubular epithelial cells expressed FSP-1, a cytoskeleton-associated, calcium-binding protein that is normally expressed in fibroblasts, but not in epithelial cells, in a mouse model of anti-tubular basement membrane disease and firstly demonstrated the presence of EMT in renal fibrosis using FSP-1 as a marker. Subsequently, Iwano *et al* (24) demonstrated that up to 36% of all FSP-1-positive fibroblasts within the interstitial space originate from renal proximal tubules following UUO, clearly confirming the significant contribution of EMT to the pathogenesis of kidney fibrosis in the model of UUO. Studies have demonstrated that tubular epithelial cells *in vitro* undergo phenotypic conversion after being incubated with TGF-β1 and that tubular epithelial cells transdifferentiate into myofibroblasts (25,26). EMT can be regulated by a number of factors in different ways; however, TGF-β1 is the most potent inducer that is capable of initiating and completing the entire EMT course (8). Thus, the mouse model of UUO and the tubular epithelial cell model stimulated by TGF-β1 are classic *in vivo* and *in vitro* renal EMT models (24,27). In addition, EMT in renal fibrosis is generally identified by the loss of epithelial proteins, including E-cadherin, ZO-1 and cytokeratin (28,29), and the acquisition of new mesenchymal markers, including vimentin, α-SMA and FSP-1 (30–32).

Li *et al* (11) reported that the hypermethylation of the KLF4 promoter mainly resulted in the inhibition of its expression in renal cancer and the overexpression of KLF4 suppressed renal cancer cell migration and invasion by altering EMT-related factors. In the present study, we investigated the expression and methylation status of KLF4 in renal EMT models *in vivo* and *in vitro*. We found that the KLF4 mRNA and protein levels were decreased in the renal tissues of mice subjected to UUO and in tubular epithelial HK-2 cells stimulated with TGF-β1, and illustrated that the reduced expression of KLF4 in renal EMT models *in vivo* or *in vitro* was accompanied by the hypermethylation of the KLF4 promoter that may lead to lower transcript levels of KLF4. *In vitro*, the reduced KLF4 expression accompanied by a decrease in E-cadherin expression and an increase in α-SMA expression were observed in the HK-2 cells stimulated with TGF-β1. However, the demethylating agent, 5-aza-2′-deoxycytidine, attenuated the TGF-β1-induced downregulation of KLF4 and E-adherin and upregulation of E-cadherin in the HK-2 cells. It is thus verified that KLF4 is hypermethylated and downregulated in the renal EMT process. These data also suggest that KLF4 functions as a suppressor of EMT induced by TGF-β1 in tubular epithelial cells.

It has been demonstrated that epienetic alterations of functional genes are associated with DNA methyltransferase (17) or histone deacetylase (14). Our results revealed that an increased Dnmt1 and Dnmt3 expression was detected in the HK-2 cells stimulated with TGF-β1. It was confirmed that Dnmt1 directly participates in the TGF-β1-mediated hypermethylation of the KLF4 promoter in HK-2 cells. It has been demonstrated that Dnmt1 catalyzes DNA methylation and subsequently leads to the transcriptional repression of genes associated with fibrogenesis; for example, the induction of RASAL1 hypermethylation contributes to renal fibroblast activation (17), the mediation of PTEN hypermethylation (33) and the epigenetic repression of Smad7 (34) in liver fibrosis. Our data provide evidence supporting the hypothesis that an increased Dnmt1 expression induced by TGF-β contributes to the epigenetic repression of KLF4 in EMT in tubular epithelial HK-2 cells.

To confirm that KLF4 functions as a suppressor of EMT in renal fibrosis, the expression of KLF4 was enforced in the HK-2 cells. The results revealed that the overexpression of KLF4 induced an increase in E-cadherin and ZO-1 expression and a decrease in a-SMA and FSP-1 expression, and attenuated the TGF-β1-induced downregulation of E-cadherin and ZO-1 expression and the upregulation of α-SMA and FSP-1. These data confirm that KLF4 inhibits the TGF-β1-induced EMT progression in renal epithelial cells, and suggest that KLF4 plays a suppressive role in EMT in renal fibrosis.

In conclusion, in this study, we demonstrate that KLF4 is downregulated in a renal EMT model *in vivo* and *in vitro*, and that KLF4 functions as suppressor of renal fibrogenesis and the hypermethylation of KLF4 mediated by Dnmt1. The downregulatin of KLF4 contributes to the progression of EMT in renal epithelial cells. Future studies are required to elucidate the utility of methylated KLF4 as a diagnostic marker or therapeutic target in renal fibrosis.
